# Mortality Benefit of Remdesivir in COVID-19: A Systematic Review and Meta-Analysis

**DOI:** 10.3389/fmed.2020.606429

**Published:** 2021-01-27

**Authors:** Vikas Bansal, Kiran S. Mahapure, Abhishek Bhurwal, Ishita Gupta, Sahar Hassanain, Janaki Makadia, Nimisha Madas, Paige Armaly, Romil Singh, Ishita Mehra, John C. O'Horo, Rahul Kashyap

**Affiliations:** ^1^Department of Anaesthesiology and Critical Care Medicine, Mayo Clinic, Rochester, MN, United States; ^2^Senior Resident, Department of Plastic Surgery, KAHER J. N. Medical College, Belgaum, India; ^3^Department of Gastroenterology and Hepatology, Rutgers Robert Wood Johnson School of Medicine, New Brunswick, NJ, United States; ^4^Dr. Rajendra Prasad Government Medical College, Tanda, India; ^5^Women Medical College, Abbottabad, Pakistan; ^6^Gujrat Medical Education and Research Society Medical College, Vadodara, India; ^7^Mamata Medical College, Khammam, India; ^8^University of the West Indies, Nassau, Bahamas; ^9^Departments of Medicine, Metropolitan Hospital, Jaipur, India; ^10^Division of Pulmonary, Sleep and Critical Care Medicine, Mayo Clinic, Rochester, MN, United States; ^11^Department of Internal Medicine, North Alabama Medical Center, Florence, AL, United States; ^12^Department of Infectious Disease, Department of Anesthesiology and Critical Care Medicine, Mayo Clinic, Rochester, MN, United States

**Keywords:** Remdesivir (GS-5734), COVID-19, SARS-CoV-2, mortality, systematic review, meta-analysis

## Abstract

**Importance/Background:** During current public health emergency of COVID-19 pandemic, repurposing of existing antiviral drugs may be an efficient strategy since there is no proven effective treatment. Published literature shows Remdesivir has broad-spectrum antiviral activity against numerous RNA viruses and has been recently recognized as a promising therapy against SARS-CoV-2.

**Methods:** A systematic search was conducted for full length manuscripts published between inception and July 19th, 2020 focussing on efficacy and safety of Remdesivir in COVID-19. The primary outcomes were defined as mortality rate and median days to recovery based on the available pooled data. The secondary outcome was adverse events rate and drug discontinuation rate.

**Statistical Analysis:** All outcomes were performed using Comprehensive Meta-Analysis software package (Bio stat, Englewood, NJ, USA).

**Results:** A total of 1,895 patients from 9 studies were included in this qualitative synthesis. In patients treated with Remdesivir, the mean recovery time was 15.84 days (95% CI 11.68–20, SE 2.12; *I*^2^ = 97.24) and the pooled mortality rate was 11.3% (95% CI 7.9–16%; *I*^2^ = 74.85). However, treatment with Remdesivir was associated with adverse effects (55.3%, 95% CI 31.5–76.9%; *I*^2^ = 97.66) eventually warranting the discontinuation of the drug (17.8%, 95% CI 8.6–33.1%; *I*^2^ = 95.64). The meta-analysis of three clinical trials indicated that administration of Remdesivir significantly reduces the mortality compared to the placebo (OR 0.70, 95% CI 0.58–0.84, *p* ≤ 0.001; *I*^2^ = 16.6).

**Conclusions and Relevance:** The result of contemporary meta-analysis suggests mortality benefit with Remdesivir in COVID-19 and median recovery time was over 2 weeks. The pooled mortality with Remdesivir was found to be very low, and this analysis can shed light on this potential treatment for COVID-19 patients.

## Highlights

**What We Already Know About This Topic**

- COVID-19 is global pandemic and Remdesivir is emerging as a promising therapeutic drug.
- Preliminary clinical trial results propose that there may be a satisfactory safety profile and better clinical outcome for Remdesivir group compared with placebo or standard of care; however, data is limited at the current time.

**What This Article Tells Us That Is New**

- Our systematic review and meta-analysis provides a detailed overview of existing literature on Remdesivir in COVID-19 to evaluate the mortality benefits and adverse events.

## Introduction

The city of Wuhan in China initially noted and reported the first case of coronavirus, termed as SARS-CoV-2, in December 2019 (1). The World Health Organization (WHO) confirmed the coronavirus outbreak as a worldwide public health emergency on January 30th, 2020, and a pandemic on March 11th, 2020 (2). The WHO estimated that significant illness could happen in as high as 13.8%, and as high as 6.1% could be serious (3). The 2019–2020 pandemic has infected more than 12 million people (4). This has resulted in more than 550,000 fatalities and correspond to a crude case mortality rate of ~4.57% (4, 5).

In current medical and public health emergency, the rapid detection of effective treatment option against SARS-CoV-2 remains challenge. The utilization of existing antiviral drugs and screening of available databases could be considered as an efficient strategy. Remdesivir, an antiviral drug, has been recently recognized as a potential therapy against SARS-CoV-2 (6, 7). On April 21st, 2020, “Solidarity,” an international clinical trial, was launched by the World Health Organization (8). The aim of the study is to compare four treatment options, including Remdesivir, to find effective therapies. On May 1st, 2020, the U.S. Food and Drug Administration allowed the emergency use of Remdesivir for the management of COVID-19 in critically ill hospitalized patients (9).

Given the limited information known about Remdesivir in COVID-19, our systemic review and meta-analysis provide a detailed overview of existing literature on Remdesivir in COVID-19 to evaluate the benefits and adverse events. This may help plan future randomized controlled trials with an appropriate size to establish the efficacy and safety of Remdesivir.

## Methods

### Search Method and Strategy

**Figure 1:**
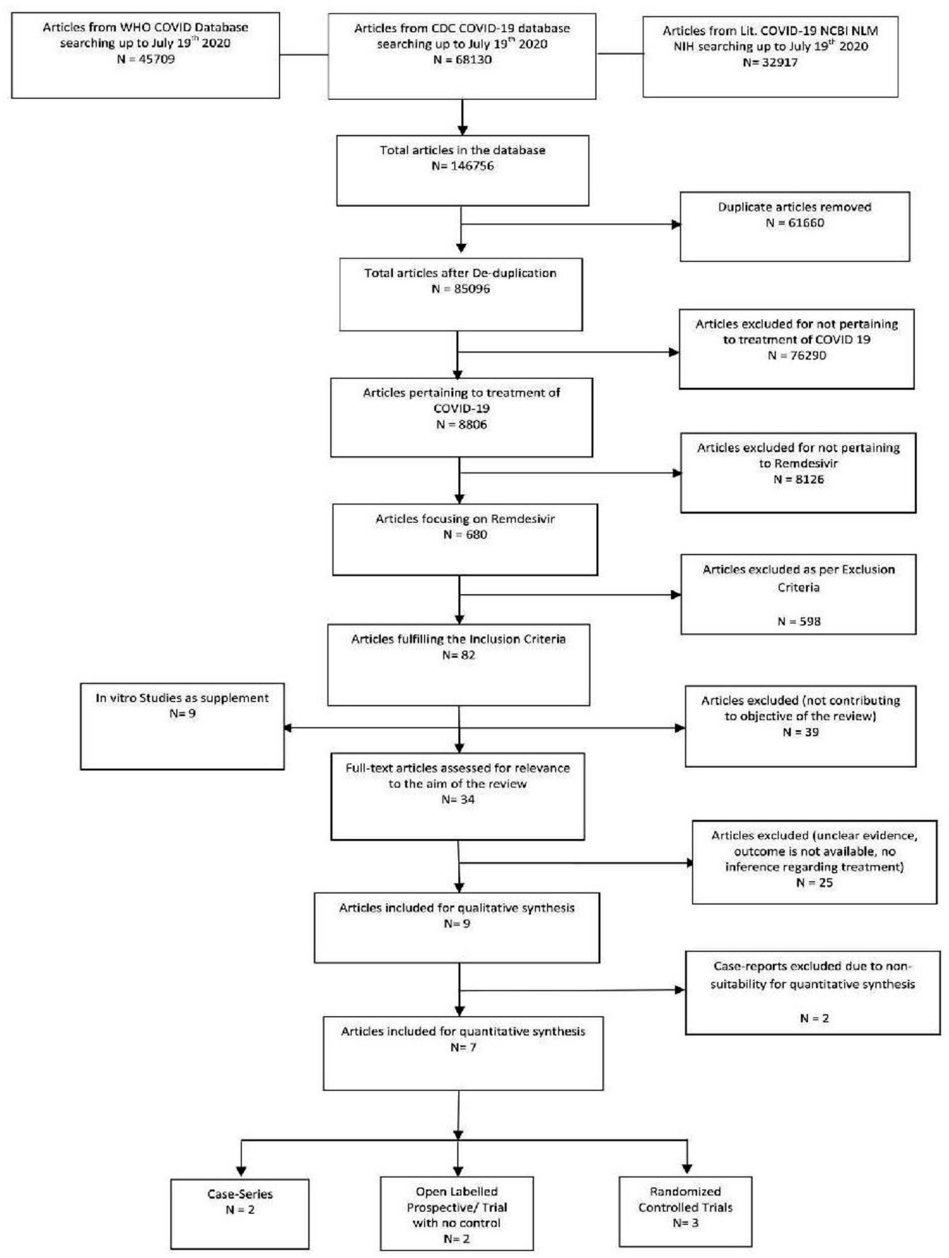
PRISMA study flow diagram.

A systematic search was conducted from COVID19 inception through July 19th, 2020, for full-length articles focusing on the efficacy and safety of Remdesivir in COVID-19. The search strategy consisted of keywords “Remdesivir,” “SARS-CoV-2,” and “COVID-19” across the COVID 19 database provided by WHO Global Research Database, CDC COVID-19 Research Articles Downloadable Database, and LitCovid database. All available databases were automatically searched from inception through July 2020 for articles describing the outcomes of COVID-19 which include Medline (Ovid and PubMed), Embase, Academic Search Complete, CAB Abstracts, Africa Wide Information, Global Health, ProQuest Central, PsycInfo, Cochrane Library, Scopus, the Virtual Health Library, CINAHL, SciFinder, and LitCovid. Other literature sources such as the Euro surveillance, China CDC Weekly, Homeland Security Digital Library, ClinicalTrials.gov, bioRxiv (preprints), medRxiv (preprints), chemRxiv (preprints), and SSRN (preprints) were searched as well. After a thorough search was performed, full-length articles meeting the inclusion criteria were evaluated. Subsequently, a manual search of the references of the included articles was accomplished. Reporting Items for Systematic Reviews and Meta-Analysis (PRISMA) guidelines were used [(10); Figure 1].

The inclusion criteria for the systematic review are as follows:

1. Studies reporting outcomes such as mortality and adverse events of Remdesivir in hospitalized patients with COVID-19.
2. Full text, peer-reviewed articles (Meta-analysis, case-studies and case series, systematic reviews, randomized controlled trials).

Once the studies met the inclusion criteria, each study was reviewed by two reviewers (KM and IG) independently, and disagreements were discussed amongst all author-reviewers and resolved via a consensus. Subsequently, the data was collected and tabulated using Microsoft Excel. The included data was checked for accuracy by all authors. Studies focussing on patients <18 years of age, pregnant females, and studies limited to particular co-morbidities and organ dysfunctions were excluded to avoid selection bias. Also, *in-vitro* studies not pertaining to the efficacy of Remdesivir in COVID-19 were excluded from quantitative synthesis (Supplementary Table 1). The data was extracted from publicly available studies; thus, IRB approval was not necessary.

### Outcomes

Primary outcomes were defined as Mortality benefit for the patients on Remdesivir in COVID-19. The mortality rate was evaluated in comparison to the control group (placebo or no Remdesivir). The defined secondary outcome was pooled adverse events rate, pooled mortality rate, the median time to recovery, and pooled drug discontinuation rate.

### Statistical Analysis

All outcomes were analyzed utilizing the Comprehensive Meta-Analysis software package (Biostat, Englewood, NJ, USA) (11). The final pooled risk estimates were obtained using random effects models (12). Raw data for events and non-events from each study were used to calculate crude odd's ratio (OR) for each study with respective 95% confidence intervals (CI) by using a random-effects model (12). To evaluate continuous outcomes, the median was converted to mean by a previously validated method (13). Subsequently, the estimates for mean recovery time were obtained using the random effects model. The Cochrane *Q* and the *I*^2^ statistics were evaluated to estimate heterogeneity between studies (14). *P* < 0.10 for the chi-square test and *I*^2^ < 20% were interpreted as low-level heterogeneity (14). The possibility of publication bias was estimated using funnel plots and with Egger's test (15).

### Risk of Bias

Two authors (KM and IG) independently assessed the risk of bias of each study included. All disagreements were discussed with all the authors, and the decision was made via a consensus. Cochrane tool for Risk of Bias (16) was used for Randomized controlled trials, and Correlation of Quality measures with estimates of treatment effects in meta-analyses of randomized controlled trials tool (17) was used for quality assessment of the same (Table 1A: Quality assessment and Risk of Bias assessment for RCT).

**Table 1A:** Quality assessment for RCT studies.

**Study ID**	**Biegel et al**.	**Wang et al**.	**Hsu et al**.	**Goldman et al**.	**Antinori et al**.
DOI	10.1056/NEJMoa2007764	10.1016/S0140 6736(20)31022-9	10.1101/2020.05.02.20088559	10.1056/NEJMoa2015301	10.1016/j.phrs.2020.104899
**Correlation of quality measures with estimates of treatment effects in meta-analyses of randomized controlled trials**					
Study question well-defined in introduction and methods	Adequate	Adequate	Adequate	Adequate	Adequate
Study question well defined anywhere in the article	Adequate	Adequate	Adequate	Adequate	Adequate
Placebo control	Adequate	Adequate	Not Defined	Not described	Not Defined
Appropriate outcome studied	Adequate	Adequate	Adequate	Adequate	Adequate
Multicenter Study	Multicenter	Multicenter	Single-center	Multicenter	Single-center
Study country	United States (45 sites), Denmark (8), the United Kingdom (5), Greece (4), Germany (3), Korea (2), Mexico (2), Spain (2), Japan (1), and Singapore (1).	China	Taiwan	United States, China, France, Germany, Hong Kong, Italy, Japan, Korea, the Netherlands, Singapore, Spain, Sweden, Switzerland, Taiwan, and the United Kingdom.	Italy
Adequate selection criteria	Adequate	Adequate	Adequate	Adequate	Adequate
Randomization methods described	Not described	The permuted block (30 patients per block) randomization sequence, including stratification, was prepared by a statistician not involved in the trial using SAS software, version 9.4	Not described	Randomized	Not described
Central randomization site	Not described	Not described	Not described	Not described	Not described
Allocation concealment	Adequate	Adequate	Inadequate	Not described	Inadequate
Patients blinded	Yes	Yes	Not described	Not described	Not described
Caregivers blinded	Yes	Yes	Not described	Not described	Not described
Outcome assessors blinded	Not described	Not described	Not described	Not described	Not described
Data analysts blinded	Yes	Not described	Not described	Not described	Not described
Double blinded	Yes	Yes	Not described	Not described	Not described
Vital statistical measures	Adequate	Adequate	Not described	Not described	Adequate
Statistician author or acknowledged	Yes	Yes	Not described	Not described	Not described
Intention-to-treat analysis	Yes	Yes	Not described	Not described	Not described
Power calculation reported	Yes	Not described	Not described	Not described	Not described
Stopping rules described	Yes	Yes	Not described	Not described	Not described
Baseline characteristics reported	Yes	Yes	Not described	Not described	Yes
Groups similar at baseline	Yes	Yes	Yes	Yes	Yes
Confounders accounted for	Not described	Not described	Not described	Not described	Not described
Percentage dropouts	Not described	10%	Not described	Not described	27%
Reasons for dropout given	Yes	Not described	Not described	Not described	Yes
Findings support conclusion	Yes	Yes	Yes	Not described	Yes
**Risk of bias as per “cochrane modified cochrane risk of bias tool” 2019**					
Random sequence generation (selection bias)	Medium risk	Low risk	Low risk	Low risk	Medium risk
Allocation concealment (selection bias)	Low risk	Low risk	Low risk	Unclear risk	Medium risk
Selective reporting (reporting bias)	Medium risk	Low risk	Low risk	Medium risk	Low risk
Other sources of bias (other bias)	Low risk	Low risk	Low risk	Low risk	Low risk
Blinding (participants and personnel) (performance bias)	Low risk	Low risk	Low risk	High risk	Low risk
Blinding (outcome assessment) (performance bias)	Low risk	Low risk	Medium risk	High risk	Low risk
Incomplete outcome data (attrition bias)	Low risk	Low risk	Low risk	Low risk	Low risk
Overall	The study is judged to raise some concerns in at least one domain for this result, but not to be at high risk of bias for any domain.	The study is judged to be at low risk of bias for all domains for this result.	The study is judged to raise some concerns in at least one domain for this result, but not to be at high risk of bias for any domain.	The study is judged to have some concerns for multiple domains in a way that substantially lowers confidence in the result.	The study is judged to have some concerns for multiple domains in a way that substantially lowers confidence in the result.

Non-randomized studies were evaluated using the NIH Quality Assessment Tool for Case Series Studies (18). Quality assessments were conducted independently, and discrepancies were resolved by consensus (Table 1B: Quality Assessment of Case series).

**Table 1B:** NIH quality assessment tool for case series studies.

**Study ID**	**Grein et al**.	**Kajawski et al**.
1. Was the study question or objective clearly stated?	Yes	Yes
2. Was the study population clearly and fully described, including a case definition?	Yes	Yes
3. Were the cases consecutive?	Yes	Yes
4. Were the subjects comparable?	Yes	Yes
5. Was the intervention clearly described?	Yes	Yes
6. Were the outcome measures clearly defined, valid, reliable, and implemented consistently across all study participants?	Yes	Yes
7. Was the length of follow-up adequate?	Yes	Yes
8. Were the statistical methods well described?	N/A	N/A
9. Were the results well described?	Yes	Yes
Quality rating (Good, Fair, and Poor)	Good	Good

## Results

### Search Results

The initial library search identified potentially relevant citations from PubMed, Medline, CENTRAL, EMBASE, Scopus, Web of Sciences, and clinical trial registries, comprised of 1,46,756 articles. Subsequently, 61,660 duplicates were removed. Out of the remaining 85,096 articles, 8,806 were focusing on the treatment of COVID-19, out of which 680 articles described Remdesivir. A total of 82 articles fulfilled the inclusion criteria, while 598 did not. The remaining manuscripts were scrutinized further, and 48 were further excluded: 39 due to non-relevance to the objective of the manuscript while 9 being *in-vitro* studies. Out of the remaining 34 articles, 26 were additionally excluded due to unclear evidence, unavailable outcome, and no reference regarding Remdesivir treatment. Thus, 9 studies were included in their entirety as shown in the qualitative synthesis, and 7 in the quantitative synthesis (2 Case series, 3 Randomized controlled trials, and 2 open-labeled prospective studies) as 2 case reports were excluded due to non-suitability for qualitative synthesis (Figure 1).

### Study Characteristics

A total of 1,895 patients from 9 articles (6, 19–26) were included in qualitative synthesis, and 7 studies were included for quantitative synthesis. Out of these, 1,237 patients were treated with Remdesivir, and 656 were not treated with Remdesivir. Among these articles, a total of three studies compared outcomes of Remdesivir in COVID-19 with placebo treatment; two were double-blinded randomized controlled trials (19, 24) while one was a simulated two-arm controlled study (22). A randomized open-label study by Goldman et al. (20) compared outcomes of 5 days course vs. 10 days course of Remdesivir. Similarly, another, open-label study also reported clinical outcomes on the 10th and 28th day of Remdesivir treatment (26). The other included studies were 2 case series (6, 23), including the study describing the compassionate use of Remdesivir in COVID-19. The study characteristics and outcomes are mentioned in Table 2.

**Table 2:** Study characteristics and outcomes.

**Study**	**Type of study**	**Total patients**	**Treatment arm**	**Control arm**	**Defined outcome**	**Average recovery time**	**Clinical improvement**		**Days of hospitalization**	**Mortality**	**Adverse events**
Grein et al. (6)	Case series	53	53	0	Decrease of 2 points or more on 6 point ordinal scale or discharge at day 28	18 days	Discontinued for 36/53 (68%) and 8/53 (15%) worsened	20/34 [17/30–57% OF IMV, 3/4 (75%) OF ECMO]	28 days	7/53 (13%)	32/53 (60%), 12/53 (23%) serious side effects and 4/32 (8%) needed discontinuation due to organ failure
Kujawski et al./COVID-Investigation Team (23)	Case series	12 (7 hospitalized)	3	4	Recovery in clinical symptoms and maintaining SpO2 above 94	Mean 14 days (6–37 days)	Not applicable	Not applicable	Mean 14 days (6–37 days)	Not mentioned	All patients had transient gastrointestinal symptoms, including nausea, vomiting, gastroparesis or rectal bleeding
Holshue et al. (21)	Case report	1	1	0	Clinical improvement and radiological findings resolution	2 days	Discontinued (for 1/1 100%) maintained spO2-96%	NA	<28 days	0	0
Hillaker et al. (25)	Case report	1	1	0	Clinical improvement and radiological findings resolution	2–4 days	Discontinued (for 1/1 100%)	1/1 (100%) extubated	<28 days	0	0
Wang et al. (24)	RCT	236	158	78	Clinical improvement up to day 28, defined as decline of two levels on a six-point ordinal scale of clinical status or discharged alive from hospital, whichever came first	Mean 19 days in treatment group vs. 21 days in control group	Discontinued for 88% in treatment group and 83% in control group	4/6 (67%) in treatment group and 1/4 (25%) in control group extubated	Mean 25 days in treatment group and 24 days in control group	22 (14%) in treatment arm vs. 10 (13%) in control group	102 (66%) in treatment arm vs. 50 (64%) in the control group, 18(12%) of Remdesivir group, and 4(5%) of control group needed discontinuation due to organ failure
Hsu et al. (22)	RCT	106	53	53	Reduction in mortality and increase in probability of discharge	5.5, 16.5, and 29.5 days for low-, medium-, and high-risk state	Not mentioned	Not mentioned	5.5, 16.5, and 29.5 days for low-, medium-, and high-risk state	7/53 (13.2%)	Not mentioned
Beigel et al. (19)	RCT	1,059	538	521	Time to recovery, defined by either discharge from the hospital or hospitalization for infection control purposes	Mean 11 days for cases vs. 15 days for the placebo group (*P* < 0.001)	Not mentioned	Not mentioned	31% shorter in treatment arm than in those who received placebo, with a 4-day reduction in hospitalization time	7.1% in treatment arm vs. 11.9% with placebo	114/541 (21.1%) in the treatment arm vs. 141/522 (27.0%) in the placebo group
NCT04292899/Goldman et al. (20)	RCT	397	397	0	Two or more points improvement in ordinal scale	14 days	Not mentioned	Not mentioned	14 days	37/397 (9.31%)	286/396 (72.04%), 29 needed discontinuation
Antinori et al. (26)	Prospective compassionate open-label study	35	35	0	Change in clinical status based on a 7-category ordinal scale	28 days follow up. Not provided mean or median recovery time	20/35discharged, nine died, three mechanically ventilated, and three improved	Not applicable	28 days	9/35	8/35 discontinued due to any adverse effects

### Dose and Treatment Regimen of Remdesivir

According to the INMI COVID-19 Treatment Group (ICOTRE Guidelines), the standard dose of Remdesivir is a loading dose of 200 mg given as an intravenous (IV) infusion over 30 min and a maintenance dose of 100 mg per day for 10 days (27). This dosing regimen was consistent with all the articles included in the analysis except one. Goldman et al. (20) compared the outcomes of a 5 vs. 10-day IV Remdesivir course in a randomized trial and found that clinical improvement on an ordinal scale was similar in both groups (*P* = 0.14).

### Primary Outcomes

#### Mortality Benefits in Remdesivir Treated Patients

Three studies described mortality in patients treated with Remdesivir compare to No-Remdesivir. Wang et al. (24) reported 28-day mortality; Beigel et al. (19) described 14-day mortality; and Hsu et al. (22) observed a statistically significant reduction of death using Remdesivir.

**Figure 2:**
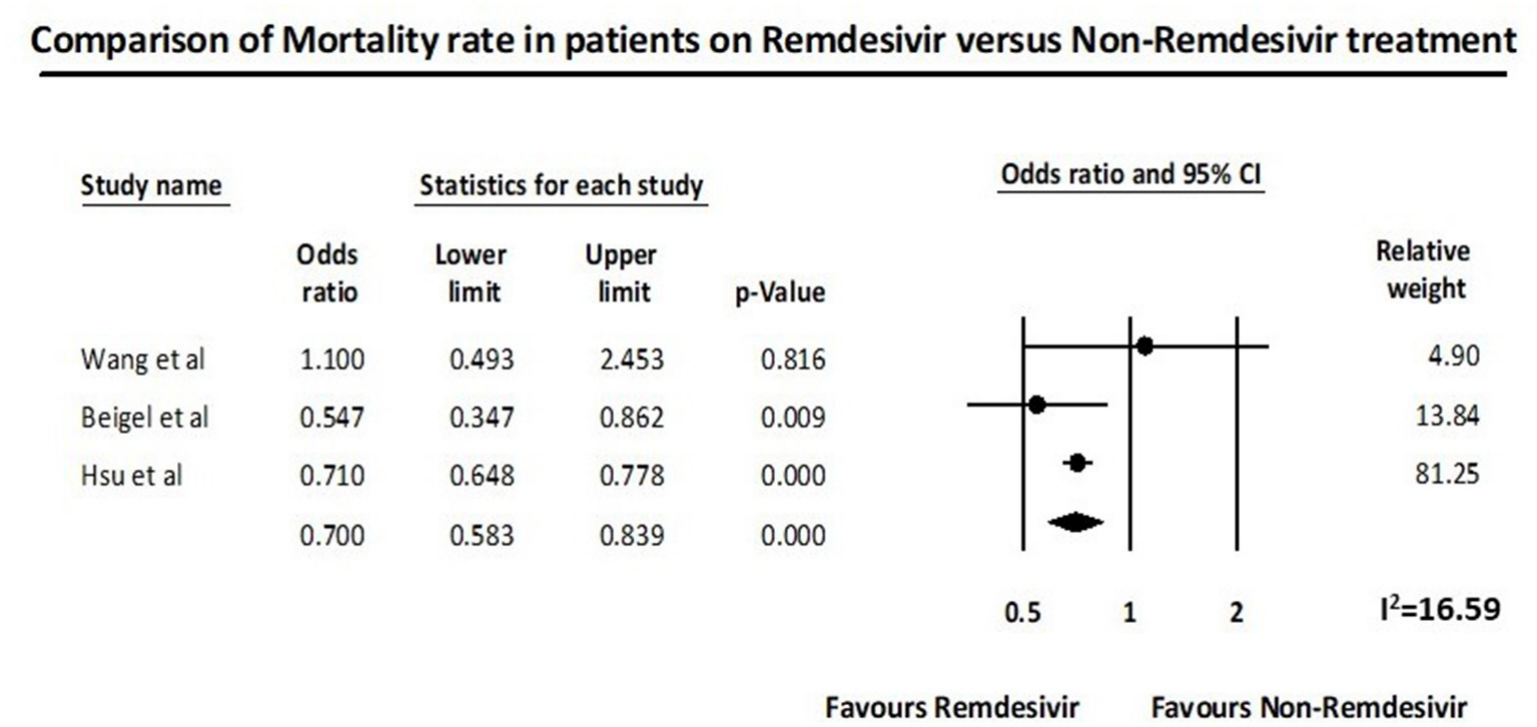
Remdesivir and mortality outcome.

The meta-analysis on the available 3 RCTs indicated that the administration of Remdesivir significantly reduces the mortality in comparison to placebo (OR 0.70, 95% CI 0.58–0.84, *p* < 0.0001; *I*^2^ = 16.59) (Figure 2).

### Secondary Outcomes

#### Pooled Mortality Rate in Remdesivir Treated Patients

The pooled mortality from all studies in patients treated with Remdesivir was 11.3% (95% CI 7.9–16%; *I*^2^ = 74.85) (Supplementary Figure 1A). Wang et al. (24) reported the highest mortality rate (14%), while Beigel et al. (19) described the lowest mortality rate (7.1%).

#### Recovery Time in Remdesivir Treated Patients

In patients treated with Remdesivir, the pooled mean recovery time from five studies was 15.84 days (95% CI 11.68–20.00, SE = 2.125) (Supplementary Figure 1B). As per the studies included in the analysis, average days of hospitalization in patients treated with Remdesivir ranged between 14 and 37 days. A 4-day reduction in hospital length of stay was noted by Beigel et al. (19) with a 31% shorter recovery time in the treatment arm compared to placebo (Table 2). According to Hsu et al. (22), Remdesivir treated patients had a 33% (95% CI 28–38%) increased odds of discharge than the control group and had a shorter hospital stay (Table 2). Hsu et al. (22) also found that the severity of COVID-19 was directly associated with a longer recovery time (Table 2). The shortest recovery time was noted in the case-report by Holshue et al. (21), were within 48 h of administration of Remdesivir, the clinical condition of the patients improved remarkably. This result was also reflected by the resolution of these patient's pulmonary lesions within 48–72 h [(21); Table 2].

#### Adverse Effects of Remdesivir

The pooled adverse event rate from six studies with Remdesivir was 55.3% (95% CI 31.5–76.9%; *I*^2^ = 97.66) (Supplementary Figure 1C). Common adverse effects reported are constipation, increased total bilirubin, increased aminotransferase levels (reversible), infusion site reactions, hypoalbuminemia, hypokalaemia, anemia, thrombocytopenia, hypoglycaemia, and pyrexia. Serious adverse effects reported are multiple-organ-dysfunction syndrome, septic shock, and acute kidney injury (Table 2).

### Discontinuation Rate

The pooled Remdesivir discontinuation rate from five studies with Remdesivir was 17.8% (95% CI 8.6–33.1%; *I*^2^ = 95.64) (Supplementary Figure 1D). The reasons for discontinuation of Remdesivir were the serious adverse effects in all the cases, and no drop cases reported in any studies (Table 2).

### Publication Bias

The funnel plot along with Egger's test (as shown in Supplementary Figure 2) revealed no publication bias. However, as there were <10 studies included in the analysis; thus publication bias cannot be completely excluded.

### Quality Assessment of Included Studies

The quality of the studies was assessed using the Cochrane Risk of Bias Tool for RCTs [(16); Table 1A]. All RCTs had adequate selection criteria. Hsu et al. (22) did not report adequate concealment, whereas Goldman et al. (20) also did not describe concealment measures. Wang et al. (24) reported adequate measures of randomization. Overall, the modified risk of bias tool showed that the included studies had low to medium risk bias.

The NIH Quality Assessment Tool (18) for Case Series was used for case series (Table 1B). Both included studies reported and fully described the population with adequate follow up. Therefore, both the studies were rated as useful on the scale.

### Narrative Synthesis *in-vitro* Studies

Remdesivir is a broad-spectrum antiviral agent that demonstrated *in vitro* and *in vivo* activity against RNA viruses (Supplementary Table 1). Remdesivir has also established broad-spectrum antiviral activity against an array of RNA virus families including Coronaviridae [SARS, MERS, and other CoV (alpha-FIP, beta-MHV, SARS1, MERS, SARS-2, and delta)], Filoviridae (Flaviviridae-Marburg and Ebola, VHF), Paramyxoviridae [Paramyxovirus (Mumps and Para-influenza), Pneumovirus (RSV), Morbillivirus (Measles), and Henipavirus (Nipah, Hendra)] (28–36); Supplementary Table 2.

## Discussion

To combat the urgent medical and public health emergency due to COVID-19, the use of existing antiviral drugs based on systematic review and meta-analysis provides the most trustworthy data regarding the outcomes of Remdesivir in COVID-19. As the information about this promising drug is limited to small sample size trials and studies, we conducted a meta-analysis and systematic review to provide high-quality evidence on the outcomes of Remdesivir in COVID-19. This provides an overview of Remdesivir's *in-vitro* studies and analyses published clinical data regarding Remdesivir's use in COVID-19.This is the first systematic review and meta-analysis to provide evidence on the efficacy and safety of Remdesivir in COVID-19.

Recently, the results from the first randomized, double-blind, placebo-controlled clinical trial using Remdesivir in COVID-19 was published (24). The study suggested a non-significant reduction in the median time to clinical improvement. However, the study may have been underpowered to detect significant differences. The Adaptive COVID-19 Treatment Trial (ACTT) (19) reported a significant reduction in recovery time in the Remdesivir group as compared to the placebo group. Additionally, the study reported a decrease in mortality amongst Remdesivir cohort as compared to placebo (19). A decision was then made by the National Institute of Allergy and Infectious Diseases (NIAID) to end this trial earlier than expected due to significant benefit of Remdesivir determined in the interim analysis (37). Similar to these findings, our result also supports the published data and confirms that Remdesivir may even reduce mortality compared with placebo or standard of care and improves time to recovery.

Our analysis also suggests a lower pooled mortality rate of 11.3% (95% CI 7.9–16%, *I*^2^ = 74.85) in COVID-19 patients. One of the reasons for lower pooled mortality could be that at the beginning of the pandemic, due to potential side effects of Remdesivir, many of the most serious patients may not have been considered to treatment and later, the inclusion of the drug in treatment protocols in less severely ill, may have introduced a confounding factor as Remdesivir treated patients are less severe.

The study which contributed significantly to the mortality benefit in our meta-analysis was the study by Hsu et al. (22). They reported 29% (95% CI 22–35%) reduction in odds of mortality with Remdesivir and a 39% decrease in the risk for the combined endpoint of severe status and death compared to the control group (22). This suggests that Remdesivir might be more effective as compared to the use in Ebola (34). A possible explanation of the improved clinical outcomes with Remdesivir could be the multiple mechanisms of action such as mutagenesis, chain termination, and perturbation of natural nucleotide triphosphate pools (33, 38). This has been shown in multiple prior *in vitro* studies (Supplementary Table 2). Remdesivir has revealed antiviral and clinical effects against SARS-CoV-1 and MERS-CoV infections in various animal models (28, 29, 31, 32, 35).

Even though some studies suggested that Remdesivir could be effective at a relatively low micro molar concentration compared with its cytotoxic concentration (29, 31), the safety of the drug in humans is still uncertain. The pooled adverse event rate from all studies with Remdesivir was 55.3% (95% CI 31.5–76.9%; *I*^2^ = 97.66). Even though some patients reported severe adverse events in the Remdesivir group compared with the placebo cohort, a higher number of patients discontinued Remdesivir (24). However, it is unknown if the liver enzyme abnormalities are a consequence of the COVID-19 itself or related to the drug. However, these abnormalities were also noticed in healthy volunteers, which may indicate that Remdesivir could be the culprit. Similar to Remdesivir, other nucleoside analogs are known to lead to liver enzyme elevations (39, 40). The most frequent mechanism postulated for increase the liver enzyme elevation is the inhibition of mitochondrial DNA synthesis. The subsequent mitochondrial dysfunction leads to multiple manifestations such as liver enzyme elevation, myopathy, pancreatitis or bone marrow suppression (39, 40). Another mechanism could be via hypersensitivity reaction or the production of toxic metabolites (39). However, these elevations tend to be idiosyncratic and uncommon, whereas liver enzyme elevations are frequently described in Remdesivir cohort. We observed that the Remdesivir discontinuation rate is relatively high 17.8% (95% CI 8.6–33.1%; *I*^2^ = 95.64). The most common reason for discontinuation of the drug was worsening respiratory failure or acute respiratory distress syndrome (24). Other reasons being elevated liver enzymes (24). The adverse events rate and drug discontinuation rate should be interpreted with caution, as causality cannot be inferred.

The strengths of our study lie in the modest number of patients across the included studies. The meta-analysis relies on shared subjectivity rather than objectivity and deals with the main effects so that results can be generalized to the target population.

Despite a large number of patients in the analysis, the meta-analysis has some limitations. A limitation of our meta-analysis based on mortality rate is inherent to the methodology. Summarizing large amounts of varying information that are useful for clinical outcomes in terms of a single number may ignore essential differences between studies. However, this limitation is a controversial aspect of meta-analysis (41). However, a meta-analysis generalizes results despite differences in primary research and does not merely report a summary effect. We observed a significant amount of heterogeneity in our studies primarily related to recovery time, pooled adverse event rate, and drug discontinuation rate. This observed heterogeneity might be due to the geographical location of the studies along with the clinical practice differences in the COVID-19 care. Another reason for heterogeneity could be the dissimilar time periods in the background of the evolving clinical evidence. The timing of Remdesivir therapy in COVID-19 may also influence outcomes, as seen in ACTT-1 trial (19). However, we were unable to pool data according to the severity of COVID-19 subgroups due to lack of available information. We would like to mention that our study predominantly describes the clinical data and incidence rates in hospitalized patients. Also the number of included studies is very few, and the analysis relied on data from case-series and clinical trials in the early phase, with a low level of evidence. Lastly, case series could also have publication bias. However, the case series were not utilized for evaluation of primary outcome and therefore less likely to influence the results overall. Even though no publication was found on visual examination of the funnel plot, further studies are needed to confirm the same. Larger scale studies (42) estimating the various systemic involvements are needed to confirm the findings.

## Conclusion

Our systemic review and meta-analysis suggest that there may be a favorable risk-benefit profile for Remdesivir compared with placebo in severe COVID-19 infection. Presently, there are no pharmacologic therapies that have shown significant benefit in COVID-19. The present COVID-19 management strategy is focused on providing supportive care and preventing complications (43, 44). Effective agents are, therefore, urgently required to relieve the burden on healthcare systems. The larger observational studies (42) and clinical trials are warranted to confirm these findings (Supplementary Table 3).

## Data Availability Statement

The original contributions presented in the study are included in the article/Supplementary Materials, further inquiries can be directed to the corresponding author/s.

## Author Contributions

KM and VB contributed equally in the defining the study outline and manuscript writing. Data review and collection done by KM, IG, SH, JM, and NM. Statistical analysis was done by VB and AB. Study design and critical review done by IM and RK. VB, KM, and AB were guarantor of the paper, taking responsibility for the integrity of the work as a whole, from inception to published article. All authors contributed to the article and approved the submitted version.

## Conflict of Interest

The authors declare that the research was conducted in the absence of any commercial or financial relationships that could be construed as a potential conflict of interest.

## Abbreviations

ACTT: the Adaptive COVID-19 Treatment Trial
ALT: alanine aminotransferase
AST: aspartate aminotransferase
CI: confidence Interval
COVID 19: Coronavirus Disease 2019
DNA: deoxyribonucleic acid
FDA: Food and Drug Administration
HIV: Human Immunodeficiency Virus
HR: Hazard Ratio
IV: intravenous
MERS: Middle Eastern Respiratory Syndrome
MERS-CoV 1: Middle Eastern Respiratory Syndrome Coronavirus 1
NJ: New Jersey
NIH: National Institute of Health
NAID: National Institute of Allergy and Infectious Diseases
OR: Odds Ratio
PRISMA: Preferred Reporting Items for Systematic Reviews and Meta-Analysis
PREVAIL study: Partnership for Research on Ebola Virus in Liberia study
PALM study: PAmoja TuLinde Maisha study
RNA: ribonucleic acid
RCT: Randomized Clinical Trial
RSV: Respiratory Syncytial Virus
SARS-CoV-2: Severe Acute Respiratory Syndrome Coronavirus 2
SARS-CoV-1: Severe Acute Respiratory Syndrome Coronavirus 1
USA: United States of America
VHF: Viral Hemorrhagic Fever
WHO: World Health Organization.
