# Supplementary material for: Mortality Benefit of Remdesivir in COVID-19: A Systematic Review and Meta-Analysis
Source: Front Med (Lausanne). 2021 Jan 27;7:606429. doi: 10.3389/fmed.2020.606429 (PMC7873594; doi:10.3389/fmed.2020.606429)
Supplement: Supplementary file 1 [file Table_1.docx]

**Table S1: Remdesivir in-vitro studies**

| **Virus** | **Author** | **DOI** | **Year** | **Country** | **Study design** | **Agent studied** | **Study methodology** | **Study outcome** |
| --- | --- | --- | --- | --- | --- | --- | --- | --- |
| SARS-CoV-2 | [Sheahan](https://www.ncbi.nlm.nih.gov/pubmed/?term=Sheahan%20TP%5BAuthor%5D&cauthor=true&cauthor_uid=28659436) T et al | <https://dx.doi.org/10.1126%2Fscitranslmed.aal3653> | 2017 | USA | Human lung epithelial cell culture, Calu-3 2B4, human airway epithelial (HAE) cell cultures,  Animal study-  Ces1c^−/−^ mice deficient in a secreted carboxylesterase | Remdesivir | HAE cells were infected with SARS-CoV at a MOI of 0.5 in the presence of RDV for 48 hours. The antiviral efficacy was evaluated by measuring intracellular genomic and sub genomic viral RNA using qrt-PCR.  Ces1c−/− mice infected with SARS-CoV were dosed subcutaneously with 50 mg/kg once daily (QD) or 25 mg/kg twice daily (BID) dose at -1dpi (prophylactic) and +1dpi (therapeutic). Their pharmacokinetic profile was studied thereafter. | A dose-dependent reduction in replication was observed at 0.1μm with average IC_50_ values of 0.069 μm for SARS-CoV in HAE cells. Also, similar reduction was observed in the genomic and subs genomic viral RNA. Both prophylactic and early therapeutic dosing demonstrated a reduction in replication below the disease-causing threshold as well as improvement in clinical signs of disease and respiratory functions. But no such difference was observed if treatment was administered 2 days post-infection. |
| SARS-CoV-2 | Das et al | 10.26434/chemrxiv.12245270 | 2020? | India | Molecular docking study | Natural anthraquinones | Blind molecular docking studies of various natural antiviral anthraquinones were carried out with SARS-CoV-2 Mpro. By this method binding sites are identified over the surface of protein. Their results were then compared with that of remdesivir. | The estimated ∆G for remdesivir is -8.99 kcal/mol which indicates highest affinity as compared to other compounds to function as a potential inhibitor for SARS-CoV-2 Mpro. |
| SARS-CoV-2 | Wang M et al | <https://doi.org/10.1038/s41422-020-0282-0> | 2020 | China | Vero cell culture | Ribavirin, penciclovir, nitazoxanide, nafamostat, chloroquine, remdesivir, favipiravir | Vero E6 cells were infected with a MOI of 0.05 at different concentrations of the test drugs. Efficacies were evaluated by quantification of viral copy numbers in the cell by qrt-PCR and confirmed by visualization of virus nucleoprotein expression at 48 h post infection. | Remdesivir and chloroquine potently blocked the virus at low-micromolar concentration and showed high SI. For Remdesivir, EC50 = 0.77 μm; EC90 = 1.76 μm; CC50 > 100 μm; SI > 129.87. Besides the site of action for chloroquine is at both entry, and at post entry stages of the nCoV infection. |
| SARS-CoV-2 | Khan S et al | <https://doi.org/10.1080/07391102.2020.1751298> | 2020 | Pakistan | Integrated Computational Approach | Remdesivir, Daraunvir and Saquinavir, flavone, coumarin derivatives | Computational drug design methods were applied to identify Chymotrypsin-like protease inhibitors from FDA approved antiviral drugs and the in-house database of drugs. Further, MD simulation and binding free energy calculations were performed on the shortlisted drugs to evaluate their various pharmacologic properties. | 3C-like protease (3CLpro) is necessary for the initiation of the viral replication process and is thus considered an established drug target for SARS-COV-2 infections. In this study, by using the silico approach, various potential inhibitors of 3CLpro were identified, Remdesivir being one of them. |
| SARS-CoV-2 | [Elfiky](https://www.ncbi.nlm.nih.gov/pubmed/?term=Elfiky%20AA%5BAuthor%5D&cauthor=true&cauthor_uid=32119961) A | <https://dx.doi.org/10.1016%2Fj.lfs.2020.117477> | 2020 | - | Molecular docking | Sofosbuvir, IDX-184, Ribavirin, Remdesivir, Guanosine triphosphate, Uracil triphosphate, Cinnamaldehyde, and Thymoquinone. | COVID-19 rdrp model was built using homology modelling after sequence comparison to the available structures in the protein data bank. Thereafter, molecular docking was performed to test some direct-acting antiviral drugs against COVID-19 rdrp. | The percent sequence identity against SARS for COVID-19 rdrp is 90.18%. Thus, we can infer that the SARS CoV rdrp is the closest strain to the COVID-19. In the molecular docking experiment, a negligible difference of 0.95 kcal/mol is seen between the SARS and COVID-19 rdrps' binding energies for Remdesivir. Thus, Remdesivir is considered as a potent drug against SARS-cov-2 since it is bound tightly and spontaneously to its rdrp. |
| SARS-CoV-2 | Pruijssers A et al | <https://doi.org/10.1101/2020.04.27.064279> | 2020 | USA | Vero cell and Calu3 2B4 cell culture. Human lung cells, primary human airway epithelial cultures,  Animal studies- Ces1c-/- mice | Remdesivir | Vero E6 and Calu3 2B4 cells were infected with SARS-cov-2 clinical isolate and treated with a range of RDV concentrations. The viral titres were then quantified by RT-qPCR.  Ces1c-/- mice were infected with SARS1/SARS2-rdrp and were treated with 25 mg/kg RDV BID subcutaneously at one day post-infection. | In Calu3 cells, RDV inhibited SARS-cov-2 with an EC50 = 0.28 μm and EC90 = 2.48 114 μm. In Vero E6 cells RDV inhibited SARS-cov-2 with EC50 = 1.65 μm and EC90 = 2.40 μm. Thus SARS-cov-2 was more potently inhibited in Calu3 2B4 than in Vero E6 cells by RDV. In HAEs, RDV demonstrated potent antiviral activity with EC50 values of 0.0010 and 0.009 µm with a selectivity index of >1000. Infected mice when treated therapeutically with RDV show decreased viral loads in the lungs and increased pulmonary function thus emphasizing RDV as a promising agent. |
| SARS-CoV-2 | Williamson B et al | https://doi.org/10.1101/2020.04.15.043166 | 2020 | USA | Animal studies- rhesus macaque model | Remdesivir | Six adult rhesus macaques infected with SARS-cov-2 strain were given RDV at 12 hours post inoculation with 10mg/kg loading dose and 5mg/kg maintenance dose intravenous bolus injection and continued once daily through 6 days post inoculation | In the animals treated with remdesivir decreased viral replication was observed in the lower respiratory tract as early as 12hrs after the first treatment. They did not show signs of respiratory disease and had reduced pulmonary infiltrates on radiographs. At necropsy on day 7 after inoculation, lung viral loads and the damage to the lung tissue was significantly lower. |
| SARS-CoV-2 | Pizzorno A et al | https://doi.org/10.1101/2020.03.31.017889 | 2020 | France | Human reconstituted airway epithelial culture, Vero cell culture | Remdesivir, diltiazem | Antiviral potential of remdesivir monotherapy as well as in combination with diltiazem against SARS-CoV-2 was studied. | Remdesivir monotherapy showed a strong antiviral effect with IC50 of 0.98 ± 0.07 µM at 48 hpi and 0.72 ± 0.03 µM at 72 hpi. This effect was greatly improved by addition of diltiazem, inducing 68% and 50% reductions in IC50 values at 48 and 72 hpi, respectively. |
| SARS-CoV-2 | Choy K et al | https://dx.doi.org/10.1016%2Fj.antiviral.2020.104786 | 2020 | China | Vero cell culture | Remdesivir, lopinavir, emetine, homoharringtonine | The antiviral effect of the aforementioned compounds was studied in the Vero E6 cells infected by SARS-CoV-2 virus at MOI = 0.02. In addition, the combinational effect of remdesivir and emetine was also studied. | EC50 of remdesivir and emetine independently was 23.15 μM and 0.46 μM, respectively. But synergy was observed with remdesivir at 6.25 μM in combination with emetine at 0.195 μM achieving 64.9% inhibition in viral yield. |
