# Supplementary material for: Mortality Benefit of Remdesivir in COVID-19: A Systematic Review and Meta-Analysis
Source: Front Med (Lausanne). 2021 Jan 27;7:606429. doi: 10.3389/fmed.2020.606429 (PMC7873594; doi:10.3389/fmed.2020.606429)
Supplement: Supplementary file 2 [file Table_2.docx]

**Table S2: Efficacy of Remdesivir against other Viruses**

| **Author** | **Virus under study** | **DOI** | **Year** | **Country** | **Study design** | **Agent studied** | **Study outcome** |
| --- | --- | --- | --- | --- | --- | --- | --- |
| **Virus Family:** Coronaviridae | | | | | | | |
| Sheahan et al | SARS-CoV  MERS-CoV | DOI: [10.1126/scitranslmed.aal3653](https://doi.org/10.1126/scitranslmed.aal3653) | 2017 | USA | Human lung epithelial cell line Calu-3 2B4 (2B4)  Primary human airway epithelial (HAE) cell cultures  Normal human bronchiolar epithelial (NHBE) | Remdesivir | Dose-dependent reduction in viral replication was reported (1 log 10 was observed at 0.1 µM and exceeded 2 log 10 at 1 µM) as compared to the untreated controls, with average IC_50_ values of 0.069 mm (SARS-CoV) and 0.074 µM (MERS-CoV).  In NHBE, the average CC_50_ for GS-5734 was determined to be 45 µM, which is 1800-fold above the observed IC_50_ value for MERS-CoV in 2B4 cells (0.025 µM) and 600-fold above the observed IC_50_ value for MERS-CoV in HAE cells (0.074 µM) |
|  | Recombinant human CoV NL63  Recombinant bat CoV for strains HKU3, HKU5, WIV1, and SHC014 |  |  |  | Primary human airway epithelial (HAE) cell cultures |  | For Recombinant human CoV NL63,  3 log 10 reductions in virus production at 0.1 µM and undetectable virus at higher concentrations.  With 1 µM GS-5734, the infectious virus production of bat CoV was reduced by 1.5 log10 to 2 log10. |
|  | SARS-CoV |  |  |  | Mouse model |  | Prophylactic administration at 50 mg/kg QD or 25 mg/kg BID ameliorated SARS-CoV induced weight loss seen with vehicle treatment. Virus titters in the lung were significantly reduced (P < 0.05) on both 2 and 5 days’ post-infection.  Therapeutic GS-5734 substantially reduced the SARS-CoV–induced weight loss in infected animals and significantly suppressed virus lung titters. |
| Brown et al | Human CoV OC43  Human CoV 229E  Zoonotic PDCoV | <https://doi.org/10.1016/j.antiviral.2019.104541> | 2019 | USA | Human hepatoma (Huh7) cells  Porcine kidney (LLCPK1) cells | Remdesivir | RDV dose-dependent reduction for Human CoV OC43 in huh cells were reported with EC_50_ equals to 0.15 µM. A similar result was also seen for Human CoV 229E in huh with an average EC_50_ 0.024 µM. RDV was not potent against HCoV-229E in LLC-PK1 cells.  In PDCoV infected LLC-PK1 cell EC _50_ could not be determined. |
| Murphy et al. | Feline infectious peritonitis (FIP) | <https://doi.org/10.1016/j.vetmic.2018.04.026> | 2018 |  | Crandell-Rees feline kidney (CRFK) cells culture | GS-441524 | Viral replication was inhibited in CRFK cell cultures at an EC_50_ of 0.78 µM and no toxicity at 100 µM. |
| Sheahan et al | MERS-CoV | DOI: https://doi.org/10.1038/s41467-019-13940-6 | 2020 | USA | Human lung epithelial cell line.  Mouse model | Remdesivir | Potent inhibition of MERS-CoV replication with an EC_50_ of 0.09 µM on Remdesivir as compared to the respective EC_50_ values generated for LPV and RTV were 11.6 and 24.9 µM.  Reduction in viral lung titters on both 4 and 6 days post-infection, virus-induced weight loss in mice, and American thoracic society lung injury scores on prophylactic RDV dose (25 mg/kg, BID). |
| Wit et al | MERS-CoV | DOI: doi/10.1073/pnas.1922083117 | 2020 | USA | Rhesus macaque model | Remdesivir | The prophylactically treated group did not display any respiratory signs of disease in contrast to the control group (6 out of 6) and therapeutically treated group (5 out of 6). |
| Agostini et al | SARS-CoV  MERS-CoV  Murine hepatitis virus | DOI: 10.1128/mBio.00221-18 | 2018 | USA | Human airway epithelial cells  Mouse delayed brain tumour | GS-441524  GS-5734 | Mean EC50 values for both viruses (SARS-CoV and MERS-CoV) infected HAE cultures were approximately 0.86 M for GS-441524 and 0.074 M for GS-5734.  Remdesivir inhibits murine hepatitis virus (MHV) with a similar 50% effective concentration values (EC_50_) as SARS-CoV and MERS-CoV. |
| **Virus Family:** Flaviviridae | | | | | | | |
| Mulangu et al | Ebola | DOI: 10.1056/NEJMoa1910993 | 2019 | Democratic Republic of Congo | Randomized clinical trial | Remdesivir | The mortality rate of 53% in 499 patients treated with Remdesivir against the EBOV disease in contrast to the mortality rate of 75% (almost 1900 people out of 2831 confirmed cases) of non-treated infected patients |
| Warren et al | Ebola | DOI:10.1038/nature17180 | 2016 | USA | Rhesus Monkey Model | GS-5734 | Significant suppression of EBOV replication was reported with 12 days regimen once-daily IV dose of 10 mg kg Remdesivir even when treatments initiated three days post-inoculation. |
| **Virus Family:** Paramyxoviridae | | | | | | | |
| Lo et al | Nipah virus Bangladesh genotype | DOI: 10.1126/scitranslmed. aau9242 | 2019 | USA | African green monkeys | Remdesivir | Animals were inoculated with a lethal dose of the Nipah virus in both treatment and control group. Mild respiratory signs were observed in two of four treated animals, whereas all control animals developed severe respiratory disease signs. |
