# Supplementary material for: Mortality Benefit of Remdesivir in COVID-19: A Systematic Review and Meta-Analysis
Source: Front Med (Lausanne). 2021 Jan 27;7:606429. doi: 10.3389/fmed.2020.606429 (PMC7873594; doi:10.3389/fmed.2020.606429)
Supplement: Supplementary file 3 [file Table_3.docx]

**Table S3: Ongoing Clinical Trials**

| Serial Number | Name of the Trial | NCT Number | Sponsor/Collaborators | Country | Study Design | Number enrolled | Interventions | Outcome | Status |
| --- | --- | --- | --- | --- | --- | --- | --- | --- | --- |
| 1 | Multicentre, Retrospective Study of the Effects of Remdesivir in the Treatment of Severe Covid-19 Infections | NCT04365725 | • Assistance Publique - Hôpitaux de Paris | • Hôpital Cochin, Paris, France | Observational | 200 | Only Remdesivir | • Clinical course on Day 15.  • Clinical course on Day 3.  • Clinical course on Day 8  • Clinical course on Day 11.  • Clinical course on Day 29.  • Duration of treatment  • Sepsis-related Organ Failure Assessment score  • Duration without mechanical ventilation  • Mortality  • Cumulative incidence of grade 3 and 4 adverse events (AEs). | Ongoing |
| 2 | Study to Evaluate the Safety and Antiviral Activity of Remdesivir (GS-5734™) in Participants With Severe Coronavirus Disease (COVID-19) | NCT04292899 | • Gilead Sciences | Multicentric, Multinational | Interventional | 6000 | Only Remdesivir | • The Odds of Ratio for Improvement on a 7-point Ordinal Scale on Day 14  • Proportion of Participants Experiencing any Treatment- Emergent Adverse Events | Ongoing |
| 3 | Study to Evaluate the Safety and Antiviral Activity of Remdesivir (GS-5734™) in Participants With Moderate Coronavirus Disease (COVID-19) Compared to Standard of Care Treatment | NCT04292730 | • Gilead Sciences | Multicentric, Multinational | Interventional | 1600 | Remdesivir and standard of care | • The Odds of Ratio for Improvement on a 7-point Ordinal Scale on Day 11  • Proportion of Participants experiencing Treatment-Emergent Adverse Events | Ongoing |
| 4 | A Trial of Remdesivir in Adults With Mild and Moderate COVID-19 | NCT04252664 | • Capital Medical University  • Chinese Academy of Medical Sciences | • Jin Yin-tan hospital, Wuhan, Hubei, China | Interventional | 308 | Remdesivir and Remdesivir placebo | • Time to Clinical recovery Time to Clinical Recovery (TTCR)  • All-cause mortality  • Frequency of respiratory progression  • Time to defervescence (in those with fever at enrolment)  • Time to cough reported as mild or absent (in those with cough at enrolment rated severe or moderate)  • Time to Dyspnoea reported as mild or absent (on a scale of severe,  moderate, mild absent, in those with dyspnoea at enrolment rated as severe or moderate,)  • Frequency of requirement for supplemental oxygen or non- invasive ventilation  • Time to 2019-nCoV RT-PCR negative in upper respiratory tract specimen  • Change (reduction) in 2019-nCoV viral load in upper respiratory tract specimen as assessed by area under viral load curve.  • Frequency of requirement for mechanical ventilation  • Frequency of serious adverse events | Suspended |
|  |  |  |  |  |  |  |  |  |  |
| 5 | A Trial of Remdesivir in Adults With Severe COVID-19 | NCT04257656 | • Capital Medical University | • Bin Cao, Beijing, Beijing, China | Interventional | 237 | Remdesivir and Remdesivir placebo | Time to Clinical Improvement (TTCI) [Censored at Day 28]  • Clinical status  • Time to Hospital Discharge OR NEWS2 (National Early Warning Score 2) of # 2 maintained for 24 hours.  • All-cause mortality  • Duration (days) of mechanical ventilation  • Duration (days) of extracorporeal membrane oxygenation  • Duration (days) of supplemental oxygenation  • Length of hospital stay (days)  • Time to 2019-nCoV RT-PCR negativity in upper and lower respiratory tract specimens  • Change (reduction) in 2019-nCoV viral load in upper and lower respiratory tract specimens as assessed by area under viral load curve.  • Frequency of serious adverse drug events | Terminated |
| 6 | Expanded Access Treatment Protocol: Remdesivir (RDV; GS-5734) for the Treatment of SARS-CoV2 (CoV) Infection (COVID-19) | NCT04323761 | • Gilead Sciences | Multicentric, Multinational | Expanded access |  | Remdesivir | - | ongoing |
| 7 | Adaptive COVID-19 Treatment Trial (ACTT) | NCT04280705 | • National Institute of Allergy and Infectious Diseases (NIAID) | Multicentric, Multinational | Interventional | 800 | Placebo and Remdesivir | • Time to recovery  • Change from baseline in alanine transaminase (ALT)  • Change from baseline in aspartate transaminase (AST)  • Change from baseline in creatinine  • Change from baseline in glucose  • Change from baseline in haemoglobin  • Change from baseline in platelets  • Change from baseline in prothrombin time (PT)  • Change from baseline in total bilirubin  • Change from baseline in white blood cell count (WBC) with differential  • and 19 more | Ongoing |
| 8 | Treatments for COVID-19: Canadian Arm of the SOLIDARITY Trial | NCT04330690 | • Sunnybrook Health Sciences Centre  • AbbVie  • Apotex Inc. | Multicentric, Multinational | Interventional | 440 | • Drug: Lopinavir/ritonavir  • Drug: Hydroxychloroquine  • Drug: remdesivir | • Efficacy of Interventions as assessed by all-cause mortality  • Time to improvement of one category from admission  • Subject clinical status  • Change in Subject clinical status  • Oxygen free days  • Incidence of oxygen use  • Duration of oxygen use  • Incidence of new mechanical ventilation  • Duration of mechanical ventilation  • Duration of hospitalization  • Mortality  • Cumulative Incidence of Grade 3 and 4 Adverse Events (AEs) and Serious Adverse Events (SAEs) | Ongoing |
| 9 | The Efficacy of Different Anti-viral Drugs in COVID 19 Infected Patients | NCT04321616 | • Oslo University Hospital | • Andreas Barratt-Due, Oslo, Norway | Interventional | 700 | • Drug: Hydroxychloroquine  • Drug: Remdesivir  • Other: (Standard of Care) | • In-hospital mortality  • Occurrence and duration of mechanical ventilation  • Occurrence and duration of intensive care unit (ICU) treatment  • Duration of hospital admittance  • 28 Day mortality  • Viral clearance as assessed by SARS-CoV-2 PCR in peripheral blood and nasopharyngeal airway specimen  • Occurrence of co-infections  • Occurrence of organ dysfunction | Ongoing |
| 10 | Expanded Access Remdesivir (RDV; GS-5734™) | NCT04302766 | • U.S. Army Medical Research and Development Command | | Expanded access |  | Only Remdesivir | The primary objective of this study is to provide expanded access of remdesivir (RDV) for the treatment of severe acute respiratory syndrome coronavirus (SARS-CoV2) infection. | Ongoing |
| 11 | Trial of Treatments for COVID-19 in Hospitalized Adults | NCT04315948 | • Institute National de la Santé Et de la  Recherche Médicale, France | Multicentric, Multinational | Interventional | 3100 | • Drug: Remdesivir  • Drug: Lopinavir/ritonavir  • Drug: Interferon Beta-1A  • Drug: Hydroxychloroquine  • Other: Standard of care | • Percentage of subjects reporting each severity rating on a 7-point ordinal scale  • Percentage of subjects reporting each severity rating on a 7-point on an ordinal scale  • The time to discharge or to a NEWS of # 2 and maintained for 24 hours, whichever occurs first.  • Number of oxygenation free days in the first 28 days  • Incidence of new oxygen use, non- invasive ventilation or high flow oxygen devices during the trial.  • Duration of new oxygen use, non- invasive ventilation or high flow oxygen devices during the trial.  • Ventilator free days in the first 28 days  • Incidence of new mechanical ventilation use during the trial.  • Hospitalization  • Mortality  • and 14 more | Ongoing |
| 12 | Antiviral Therapy and Baricitinib for the Treatment of Patients With Moderate or Severe COVID-19 | NCT04373044 | • University of Southern California  • National Cancer Institute (NCI) | • Los Angeles County-USC Medical Center, Los Angeles, California, United States  • USC / Norris Comprehensive Cancer Center, Los Angeles, California, United States | Interventional | 59 | • Drug: Baricitinib  • Drug: Hydroxychloroquine  • Drug: Lopinavir/Ritonavir  • Drug: Remdesivir | • Proportion of patients requiring invasive mechanical ventilation or dying  • Identification of clinical features (vital signs - body temperature)  • Identification of clinical features (vital signs - respiratory rate)  • Identification of clinical features (vital signs - heart rate)  • Identification of clinical features (vital signs - blood pressure)  • Identification of clinical features (Imaging)  • Identification of clinical features (Lab - White Blood Count)  • Identification of clinical features (Lab - Absolute Lymphocyte Count)  • Identification of clinical features (Lab - Haemoglobin)  • Identification of clinical features (Lab - Creatinine)  • and 4 more | Not yet recruiting |
| 13 | Effect of Treatments in Patients Hospitalized for Severe COVID-19 Pneumonia: a Multicenter Cohort Study | NCT04365764 | • Groupe Hospitalier Pitie-Salpetriere  • Centre Hospitalier Intercommunal Robert Ballanger | • Centre Hospitalier Intercommunal Robert Ballanger, Aulnay-sous-Bois, France  • Centre Hospitalier Intercommunal Le Raincy-Montfermeil, Montfermeil, France | Observational | 400 | Remdesivir | • Composite of death and mechanical ventilation  • Death  • Mechanical ventilation  • World Health Organization score | Ongoing |
| 14 | The Fleming [FMTVDM] Directed CoVid-19 Treatment Protocol | NCT04349410 | • The Camelot Foundation | • FHHI-OI-Camelot; QME, Los Angeles, California, United States | Interventional | 500 | • Drug: Hydroxychloroquine, Azithromycin  • Drug: Hydroxychloroquine, Doxycycline  • Drug: Hydroxychloroquine, Clindamycin  • Drug: Hydroxychloroquine, Clindamycin, Primaquine - low dose.  • Drug: Hydroxychloroquine, Clindamycin, Primaquine - high dose.  • Drug: Remdesivir  • Drug: Tocilizumab  • Drug: Methylprednisolone  • Drug: Interferon-Alpha2B  • Drug: Losartan  • Drug: Convalescent Serum | • Improvement in FMTVDM Measurement with nuclear imaging.  • Ventilator status  • Survival status | Ongoing |
