# Supplementary material for: Mortality Benefit of Remdesivir in COVID-19: A Systematic Review and Meta-Analysis
Source: Front Med (Lausanne). 2021 Jan 27;7:606429. doi: 10.3389/fmed.2020.606429 (PMC7873594; doi:10.3389/fmed.2020.606429)
Supplement: Supplementary file 5 [file Data_Sheet_1.docx]

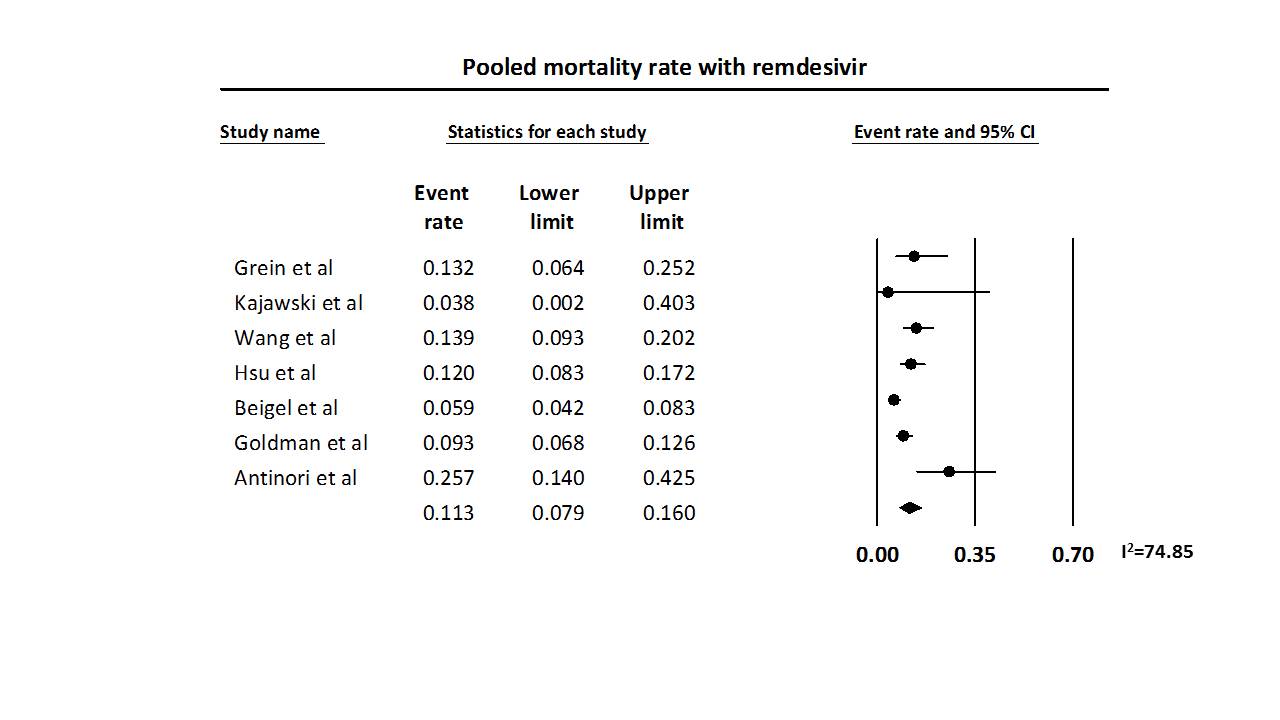
**Figure S1A: Pooled mortality with remdesivir**


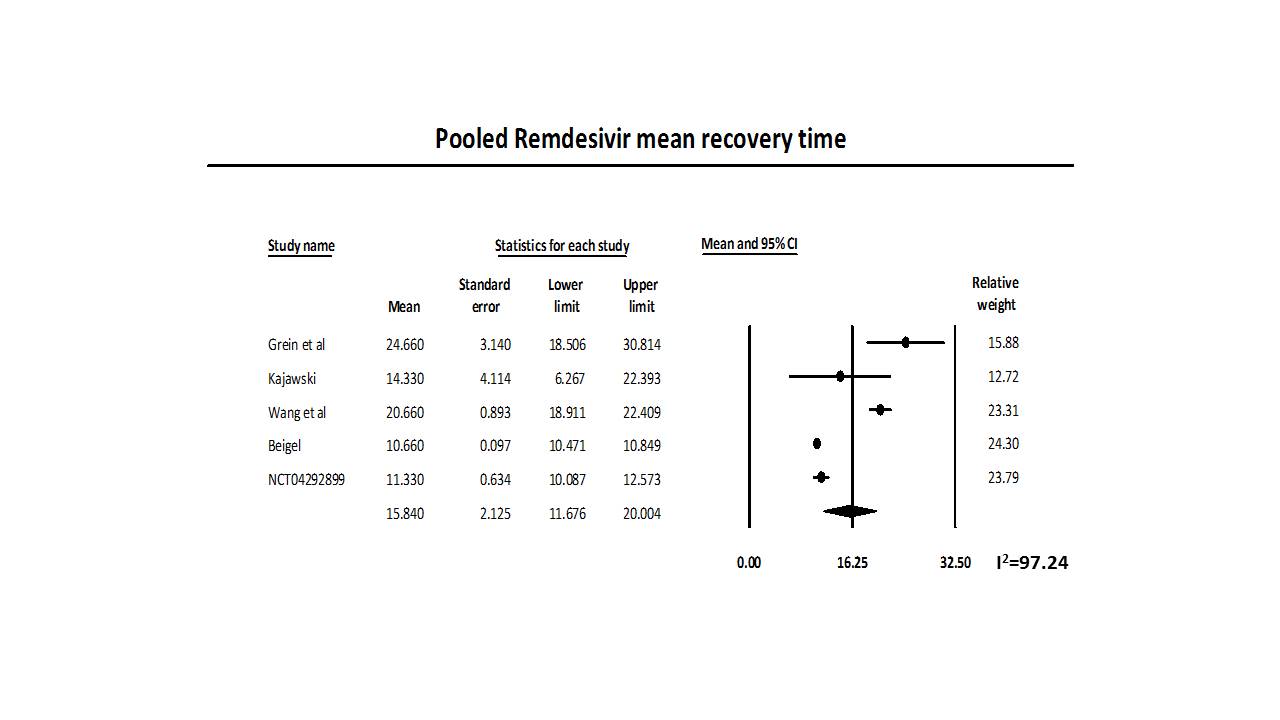
**Figure S1B: Pooled Mean Recovery time with remdesivir**

**Figure S1C: Pooled adverse event rate with remdesivir**


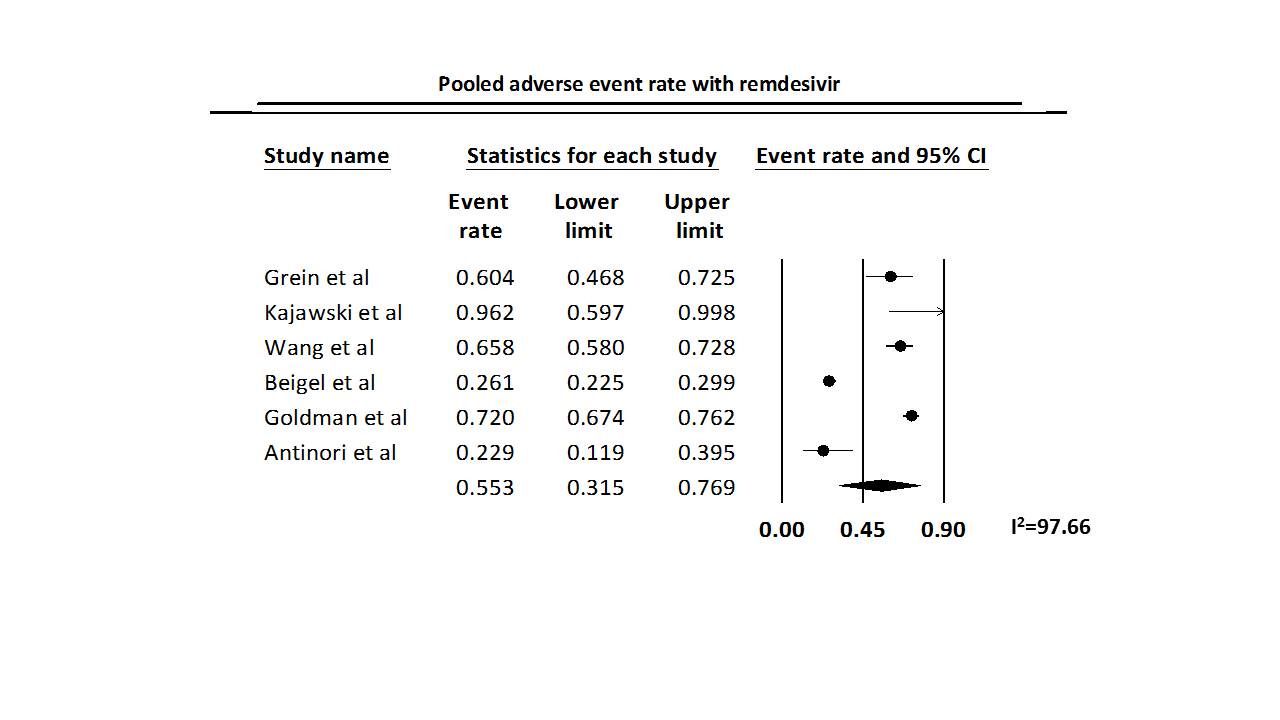


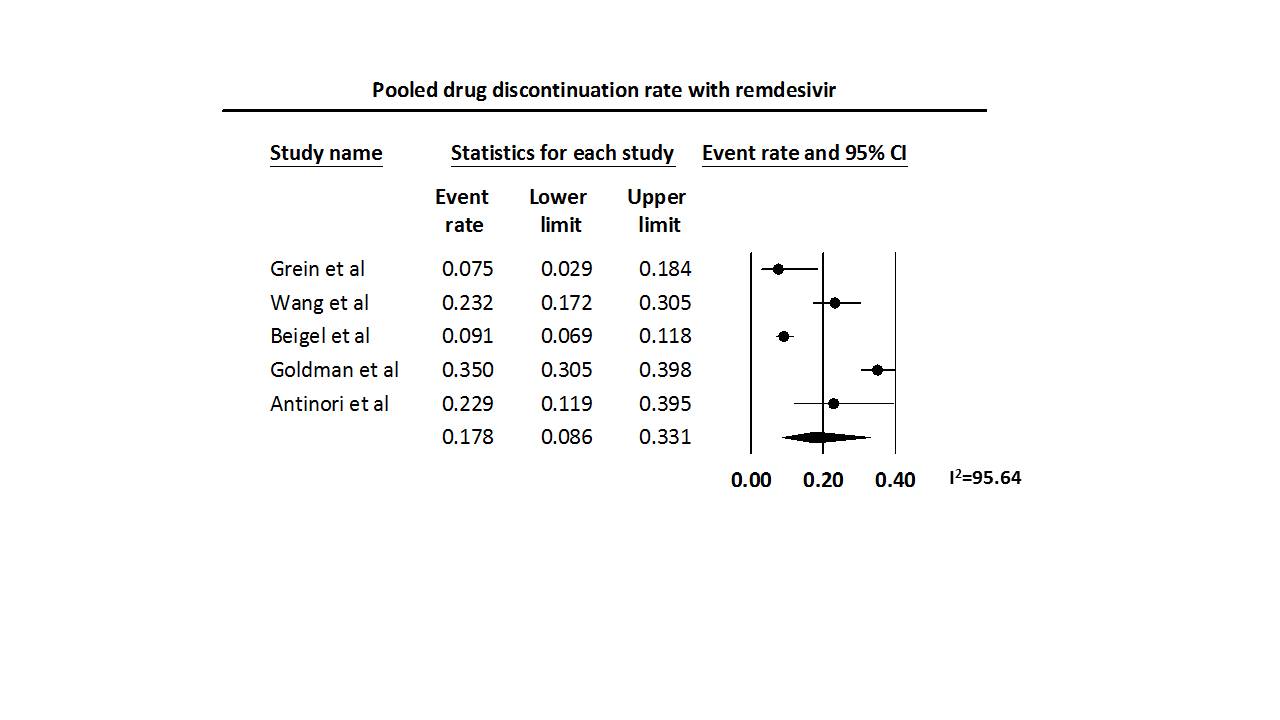
**Figure S1D: Pooled Drug discontinuation rate with remdesivir**
