# Supplementary figures and images for: Mortality Benefit of Remdesivir in COVID-19: A Systematic Review and Meta-Analysis
Source: Front Med (Lausanne). 2021 Jan 27;7:606429. doi: 10.3389/fmed.2020.606429 (PMC7873594; doi:10.3389/fmed.2020.606429)

**Figure S2: Funnel Plot of standard error by logit event rate**


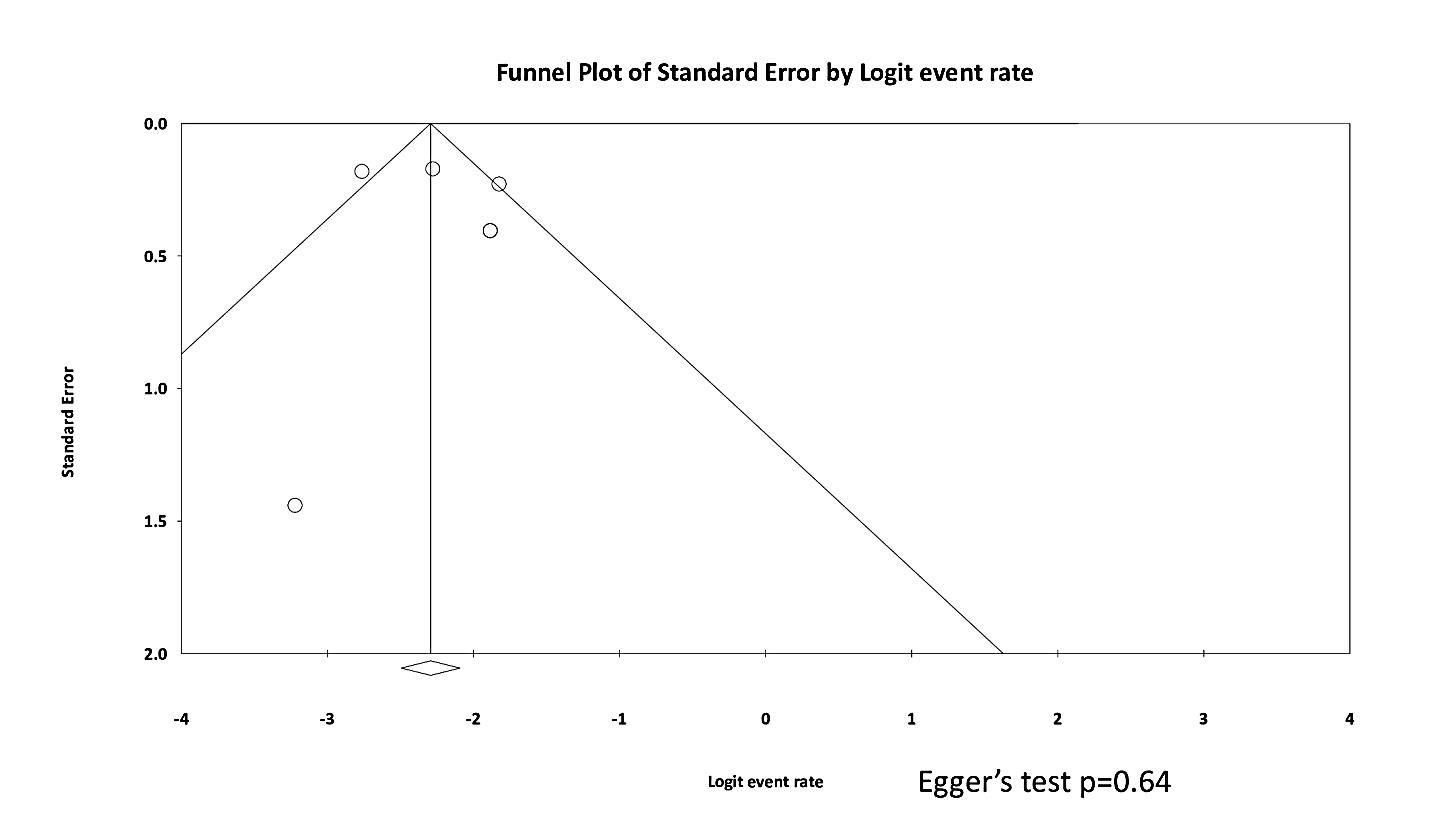

Supplement: Supplementary file 6 [file Data_Sheet_2.docx]
